# Oral Treatment of Spontaneously Hypertensive Rats with Captopril-Surface Functionalized Furosemide-Loaded Multi-Wall Lipid-Core Nanocapsules

**DOI:** 10.3390/pharmaceutics12010080

**Published:** 2020-01-18

**Authors:** Cecilia B. Michalowski, Marcelo D. Arbo, Louise Altknecht, Andréia N. Anciuti, Angélica S. G. Abreu, Luciana M. R. Alencar, Adriana R. Pohlmann, Solange C. Garcia, Sílvia S. Guterres

**Affiliations:** 1 Programa de Pós-Graduação em Ciências Farmacêuticas, Universidade Federal do Rio Grande do Sul, Avenida Ipiranga 2752, Porto Alegr 90610-000, Brazil; cbmicha@hotmail.com (C.B.M.); marcelo.arbo@gmail.com (M.D.A.); adriana.pohlmann@ufrgs.br (A.R.P.); 2 Departamento de Produção e Controle de Medicamentos, Faculdade de Farmácia, Universidade Federal do Rio Grande do Sul, Avenida Ipiranga 2752, Porto Alegre 90610-000, Brazil; 3 Laboratório de Toxicologia (LATOX), Universidade Federal do Rio Grande do Sul, Avenida Ipiranga 2752, Porto Alegre 90610-000, Brazil; lualtk@gmail.com; 4 Instituto de Ciências Básicas da Saúde, Departamento de Bioquímica, Universidade Federal do Rio Grande do Sul, Rua Ramiro Barcelos, 2600 Anexo, Porto Alegre 90035-003, Brazil; vet.andreia@gmail.com; 5 Laboratório de Microscopia Avançada, Departamento de Física, Universidade Federal do Ceara, Campus do Pici, Fortaleza 60455-900, Brazil; samara.abreu@alu.ufc.br (A.S.G.A.); lucianamagal@fisica.ufc.br (L.M.R.A.); 6 Departamento de Química Orgânica, Instituto de Química, Universidade Federal do Rio Grande do Sul, PBox 15003, Avenida Bento Gonçalves, 9500, Porto Alegre 91501-970, Brazil

**Keywords:** lipid-core nanocapsules, antihypertensive, surface-functionalization, captopril, furosemide, toxicity, oral drug delivery

## Abstract

Multi-wall lipid-core nanocapsule (MLNC) functionalized with captopril and nanoencapsulating furosemide within the core was developed as a liquid formulation for oral administration. The nanocapsules had mean particle size below 200 nm, showing unimodal and narrow size distributions with moderate dispersity (laser diffraction and dynamic light scattering). Zeta potential was inverted from −14.3 mV [LNC-Fur(0,5)] to +18.3 mV after chitosan coating. Transmission electron microscopy and atomic force microscopy showed spherical structures corroborating the nanometric diameter of the nanocapsules. Regarding the systolic pressure, on the first day, the formulations showed antihypertensive effect and a longer effect than the respective drug solutions. When both drugs were associated, the anti-hypertensive effect was prolonged. On the fifth day, a time effect reduction was observed for all treatments, except for the nanocapsule formulation containing both drugs [Capt(0.5)-Zn(25)-MLNC-Fur(0.45)]. For diastolic pressure, only Capt(0.5)-Zn(25)-MLNC-Fur(0.45) presented a significant difference (*p* < 0.05) on the first day. On the fifth day, both Capt(0.5)-MLNC-Fur(0.45) and Capt(0.5)-Zn(25)-MLNC-Fur(0.45) had an effect lasting up to 24 h. The analysis of early kidney damage marker showed a potential protection in renal function by Capt(0.5)-Zn(25)-MLNC-Fur(0.45). In conclusion, the formulation Capt(0.5)-Zn(25)-MLNC-Fur(0.45) proved to be suitable for hypertension treatment envisaging an important innovation.

## 1. Introduction

The development of effective technologies to enable the delivery of BCS (Biopharmaceutical Classification System) class IV drugs is a challenge, because the dissolution is a rate-limiting step for oral absorption [1]. Besides, these drugs present poor permeability, which causes low bioavailability. Furosemide, a class IV drug (low solubility and low permeability), is a loop diuretic used in treating hypertension and edema showing erratic and highly variable absorption and low bioavailability administered by the oral via. Nanotechnologies can offer solutions to circumvent these limitations. Solid nanodispersions improved the dissolution, flux and rat plasma exposure of furosemide, being an effective drug solubilization platform to improve the oral absorption of poorly water soluble drugs [2]. Chitosan/alginate nanoparticles containing furosemide improved its ex vivo intestinal permeability and pharmacokinetic parameters [3]. Nanosuspensions of furosemide were produced using high pressure homogenization technique using ultrasonic probe or Ultra-Turrax^®^, ball milling method, and a combination of them. Results showed the formulations are a promising approach for increasing the solubility and permeability properties of poorly water soluble drugs [4]. To the best of our knowledge, there is no report in the literature on the encapsulation of furosemide in polymeric nanocapsules, specifically in lipid-core nanocapsules. Unlike conventional nanocapsules and nanoparticles, lipid-core nanocapsules are a core-shell system, containing an inner core composed of an organogel rather than a liquid core. This feature causes two diffusional barriers for the drug release, the diffusion in the lipid-core and the diffusion through the polymeric wall. Due to this property, lipid-core nanocapsules better physically protect nanoencapsulated drugs, increase apparent solubility, and effectively control the drug release [5].

On the other hand, captopril, a class III drug (high solubility and low permeability), is used in hypertension management. However, its antihypertensive action is short and its oral bioavailability is very low and sometimes irregular. In this way, the nanoencapsulation of captopril can prolong the delivery of the drug and contribute in the reduction of the dosing frequency and in the increase of the drug bioavailability. For instance, cyclodextrin nanoparticles containing captopril exhibited long-lasting effects in controlling angiotensin I pressor effect [6].

Previous studies showed that captopril given in combination with furosemide caused a higher decrease in arterial pressure in patients compared to the drugs administered alone [7,8,9]. Although captopril and furosemide are frequently combined together in therapeutics, there is no report about their association in a unique nanocarrier. Most likely, one of the difficulties in formulating them in the same nanoparticle is their very different lipophilicities. A promising strategy to combine those drugs in a single nanocarrier could be encapsulate the class IV drug (most lipophilic) in the nanocarrier and bind the class III drug (more hydrophilic) onto the surface.

In the literature, the surface functionalization of nanocarriers is described as a strategy to increase drug uptake [10,11,12,13,14], prolong blood circulation time [15,16], and ameliorate function of active targeting [12,14,17,18,19,20] improving therapeutic efficacy. Different methods have been developed to modify the surface of the particles. Some studies describe the use of reactive linkers, like nanoparticle activation by epoxy compound [10,12], others use coupling agents, such as conjugation of poly(d,l-lactide-*co*-glycolide) (PLGA) nanoparticles with wheat germ agglutinin [21], or with folic acid, for solid tumor treatment when folate receptor is overexpressed [20,22]. A common aspect in all those approaches is the requirement of a purification step after the surface functionalization. In parallel, pre-functionalization of the nanoparticle materials, for instance the polymer, in which functional ligands are covalently conjugated, can be used as a strategy. Several studies have proposed this approach. PLGA-*block*-poly(ethyleneglycol) (PLGA-*b*-PEG), for example, was used to conjugate the A10 RNA aptamer (Apt) to bind the prostate specific membrane antigen [17,23,24]. The method requires a sequence of incubation, extractions and ultrafiltration followed by the reaction with the aptamer and its denaturation. Additionally, PLGA-*b*-PEG were used to conjugate *S*,*S*-2-[3-[5-amino-1-carboxypentyl]-ureido]pentanedioic acid (ACUPA) to the same therapeutic purpose [25] using a three step process, first attaching the ACUPA precursor followed by the purification (filtration, precipitation and drying), second the polymerization followed by a purification step (precipitation and drying) and finally removing the allyl protecting groups. More recently, PLGA-PEG was conjugated to *cyclic*-arginylglycylaspartic acid (*c*RGD) that highly binds to integrin receptors (αvβ3 and αvβ5). Nevertheless, several precipitation steps in solvents and a dialysis against water are required for purification [26]. Those approaches, however, must be performed for each specific ligand, besides they are time-consuming.

A few years ago, our research group proposed a new process to functionalize the surface of chitosan-coated lipid-core nanocapsules using a ligand-metal ion-chitosan-lecithin complex [27,28,29,30,31,32,33]. This method is based on a rapid one-pot synthesis without needing any purification step, overcoming the drawbacks of other methodologies. Our studies describing the development of multiwall nanocapsules have been published since 2014. One advantage of the multiwall lipid-core nanocapsules is that we can obtain a complex system using a simple process. The functionalization of the multiwall nanocapsules with a single-chain variable fragment [scFv anti-LDL(-)] was developed with the objective to inhibit the progression of atherosclerotic lesions. The scFv anti-LDL(-), complexed to the nanocapsule surface, retained its reactivity and specificity to recognize the electronegative low density lipoprotein [LDL(−)] [27]. LDL receptor knockout mice treated with the scFv anti-LDL(-)-nanoformulation by intravenous administration showed a decrease in the atherosclerotic lesions with no signal of acute toxicity. Furthermore, a significant downregulation of *Il1b* mRNA expression and a decrease on expression of CD14 protein have been observed for the treated group [30]. In addition, we previously determined that the chitosan-coated lipid-core nanocapsules are hemocompatible, since no significant hemolysis or platelet aggregation have been observed in vitro after adding this liquid formulation into human blood and plasma at a proportion of 1:100 (*v*/*v*) [28].

In another study, we proposed the use of laronidase as ligand on the surface of the chitosan-coated lipid-core nanocapsules [29]. Laronidase is an enzyme used in the treatment of mucopolysaccharidosis type 1, a genetic disorder caused by alpha-l-iduronidase deficiency. Laronidase forms aggregates in water and its binding to the nanocapsule surface exposed a larger number of active sites leading to a higher catalytic efficiency in vitro. The liquid formulation containing the laronidase-functionalized multiwall lipid-core nanocapsules showed a higher catalytic activity in vivo in liver, kidney and heart compared to the commercial product. The results pointed out a probable reduction in the dose of the treatment and in the use of antipyretics and antihistamines before the enzyme infusion.

More recently, we developed surface-functionalized nanocapsules using bromelain [31], a protease presenting antiproliferative effect. The enzyme maintained its proteolytic effect (in vitro) after immobilization on the nanocapsule surface; and when tested in vitro against human breast cancer cells (MCF-7), the bromelain-surface-functionalized nanocapsules showed a decrease on cell viability compared to the control. Considering the presence of folate receptors in the membrane of MCF-7 cells, we proposed the development of methotrexate-functionalized nanocapsules [32]. Methotrexate and its diethyl ester derivative have been encapsulated and/or used as ligands in the nanocapsule formulations. When the drug or the pro-drug were acting as ligands at the nanocapsule surface, a higher uptake by MCF-7 cells and a higher antiproliferative effect have been observed compared to the control or to the formulation functionalized with a non-active model of ligand (phenylalanine). In parallel, we developed doxorubicin-loaded nanocapsules having RGD as ligand on the surface to target tumoral cells overexpressing αvβ3 integrin [33]. An improved uptake of the nanocapsules by U87MG cells and a significant decrease in cell viability compared to control have been observed.

Briefly, our previous studies showed the feasibility of decorating the nanocapsule surface with proteins (macromolecules) or even low molecular weight drugs (methotrexate or its diethyl ester derivative), as well as their improved in vitro and/or in vivo biological effects. In the current study, we hypothesized that captopril and furosemide could be simultaneously vehiculated in multi-wall lipid-core nanocapsule. Captopril presents a carboxylic function able to coordinate to Zn^2+^ on the nanocapsule surface, and furosemide, having a log *D* of 1.91 at pH 4.05, could be encapsulated in the lipid-core of the nanocapsules.

Our hypothesis considered that their association in multiwall nanocapsules could bring improvements in treatment effectiveness. To prove this concept, the pharmacodynamic effect and the toxicity of this innovative formulation (captopril-surface functionalized furosemide-loaded multiwall nanocapsules) administered by the oral route to spontaneously hypertensive rats were evaluated. The study constitutes the first investigation of the surface-functionalized nanocapsules administered by the oral route.

## 2. Materials and Methods

### 2.1. Materials

Poly(ε-caprolactone) (PCL) (MW 80,000 g mol^−1^), caprylic/capric triglyceride, sorbitan monostearate (Span 60^®^) and chitosan low molar weight (MW 50,000–190,000 g mol^−1^, 75–85% deacetylated polymer) were obtained from Sigma-Aldrich (São Paulo, Brazil). Soybean lecithin (Lipoid S75^®^) was obtained from Lipoid (Ludwigshafen, Germany). Polysorbate 80 (Tween80^®^) was purchased from Henrifarma (São Paulo, Brazil) and captopril and furosemide from Delaware (Porto Alegre, Brazil). All solvents used were of analytical or pharmaceutical grades and were used as received.

### 2.2. Preparation of Captopril-Functionalized Furosemide-Loaded Nanocapsules Dispersed in Water

Captopril-functionalized furosemide-loaded nanocapsules dispersed in water was produced in batches of 10 mL. For comparative purposes, we prepared a formulation containing exclusively captopril (captopril-functionalized nanocapsules dispersed in water). The lipid-core nanocapsules were prepared by self-assembling as previously reported [28,34]. The organic phase (A) was composed by PCL (0.100 g), sorbitan monostearate (0.038 g), furosemide (0 or 0.005 g) and capric/caprylic triglyceride (0.160 mL) dissolved in acetone (25.0 mL) under heat (40 °C) and magnetic stirring. In another flask (B), soybean lecithin (0.060 g) was dissolved in ethanol (3.0 mL) and added into the organic phase (A). Polysorbate 80 (0.0776 g) was dispersed into 53.0 mL of water (C) and after all the components were dissolved, the transparent homogeneous mixture A + B was injected into the aqueous phase (C) instantaneously forming a turbid homogeneous solution. The turbid solution was kept under magnetic stirring at room temperature for 10 min. Under reduced pressure and temperature of 40 °C (Rotavapor RII, Recirculating Chiller B-740, Büchi, Switzerland), the organic solvents and part of the water were eliminated to adjust the final volume of each formulation to 10 mL. The formulations were called lipid-core nanocapsules (LNC) and furosemide-loaded lipid-core nanocapsules [LNC-Fur(0.5)].

A chitosan aqueous solution was prepared (1.0%, *w*/*v*) using 1.0% acetic acid, solution D. After chitosan was solubilized, the solution D was filtered through a 0.45 μm membrane (Millipore^®^) to avoid clusters. One milliliter of the filtered solution D was dripped over 9.0 mL of LNC [or LNC-Fur(0.5)] formulation that was kept under magnetic stirring for two hours at room temperature. The formulations were called MLNC and furosemide-loaded multi-wall lipid-core nanocapsules [MLNC-Fur(0.5)]. The last step of the preparation process was performed by the captopril complexation at the surface of the nanocapsules. An 8 mg mL^−1^ zinc acetate solution (E), 32 µL, was added into 10 mL of MLNC-Fur(0.45) under magnetic stirring and right after (1 min) 135 µL of captopril solution at 40 mg mL^−1^ was added into the medium and kept under magnetic stirring for 24 h. The formulations were named Capt(0.5)-Zn-MLNC and Capt(0.5)-Zn-MLNC-Fur(0.45). For comparison, a formulation prepared without adding zinc acetate [Capt(0.5)-MLNC-Fur(0.45)] was used in the in vivo studies.

### 2.3. Quantification of Captopril and Furosemide by High Performance Liquid Chromatography (HPLC)

#### 2.3.1. Furosemide and Captopril Analytical Methods

To evaluate the content of the drugs, the method described by Azevedo and co-workers [35] was used with modifications. The analysis was performed by High Performance Liquid Chromatography (HPLC) system consisted of a Perkin Elmer S-200 (Waltham, MA, USA) with an S-200 injector, a UV-VIS detector with UV detection at 215 nm and a flow rate of 1.0 mL min^−1^. The mobile phase was composed of 0.11% (*v*/*v*) phosphoric acid aqueous solution/methanol/acetonitrile 50:25:25 (*v*/*v*/*v*) previously filtered through a 0.45 µm membrane and degassed by sonication. The separation was performed in an analytical column 250 × 4.6 mm internal diameter C_18_, 5.0 µm particle size (Phenomenex^®^, Torrance, CA, USA). A standard solution containing both, captopril (1 mg mL^−1^) and furosemide (1 mg mL^−1^), was prepared using acetonitrile. The calibration curve was prepared by diluting aliquots of the standard solution in the mobile phase at the following concentrations of each drug: 0.5, 1.0, 2.5, 5.0, 10.0, 20.0 and 40.0 µg mL^−1^.

#### 2.3.2. Drug Contents

For the extraction of the drugs, 500.0 µL of the formulation was diluted in 10.0 mL of acetonitrile in a 25.0 mL volumetric flask, and sonicated. After 10 min, the volume was completed with 0.11% (*v*/*v*) phosphoric acid aqueous solution and sonicated once again for 10 min. The sample was filtered in a 0.45 µm membrane (Regenerated Cellulose Membrane filter, Sartorius Stedim, Göttingen, Germany) and injected for HPLC analysis.

#### 2.3.3. Furosemide and Captopril Encapsulation Efficiencies

Furosemide and captopril encapsulation efficiencies in Capt(0.5)-Zn(25)-MLNC and in Capt(0.5)-Zn(25)-MLNC-Fur(0.45) were calculated after performing a ultrafiltration-centrifugation (cut off 10 kDa, Millipore, Burlington, MA, USA) at 1976× *g* for 10 min and quantification of both drugs in the ultrafiltrate by HPLC. The concentration of the encapsulated drug was calculated by the difference between the drug content, i.e., total concentration (*C_t_*) in the formulation, and its concentration in the ultrafiltrate (*C_ultrafiltrate_*). The encapsulation efficiency (*EE*%) was obtained by dividing this difference by the drug content and multiplying by 100 (Equation (1)).

(1) $$EE\left(\%\right)=\frac{\left(C_{t}-C_{ultrafiltrate}\right)\times 100}{C_{t}}$$

To determine any interaction of the excipients and captopril with the membrane of the ultrafiltration unit, the ultrafiltration-centrifugation technique was carried out for the following compositions prepared at similar concentrations to Capt(0.5)-Zn(25)-MLNC-Fur(0.45): (i) a solution of captopril, chitosan and polysorbate 80; (ii) a solution of captopril, zinc acetate and polysorbate 80; and (iii) Capt(0.5)-MLNC-Fur(0.45).

### 2.4. Captopril and Furosemide Dialysis to Determine the Optimal Concentrations of Zn^2+^ and Captopril in the Capt(0.5)-Zn-MLNC-Fur(0.45) Formulation

To determine the optimal concentration of Zn^2+^ in Capt(0.5)-Zn-MLNC-Fur(0.45) formulation, captopril dialysis experiments were performed. Five formulations were prepared, maintaining the captopril concentration constant at 0.5 mg·mL^−1^ and varying the Zn^2+^ concentration (0, 25, 50, 100, 200 e 400 µg mL^−1^). The drug diffusion through the dialysis bag (10–12 kDa membrane, 25 mm, Sigma-Aldrich^®^, San Luis, MO, USA) was performed separately by adding 4.0 mL of each nanocapsule formulation inside each dialysis bag. Each bag was placed inside a glass recipient containing 40 mL of water. The experiment was carried out under magnetic stirring at 37 °C for 6 h. The medium was collected and replaced in pre-determined time intervals (0, 5, 1, 2, 4 and 6 h). The data (triplicate) were expressed in percentage in relation to the drug content in the nanocapsule formulation.

### 2.5. Particle Sizing

#### 2.5.1. Laser Diffraction

Analyses were carried out with three different batches of each formulation. The formulations were firstly analyzed by Mastersizer^®^ 2000 laser diffraction instrument (Malvern Instruments, Malvern, UK) to determine the particle distribution in the range between 0.02 to 2000 μm. Approximately 150 mL of distilled water was added in the Small Volume Sample Dispersion Unit (Malvern Instruments, Malvern, UK) and the background subtracted from each analysis. Each sample (450 µL) was added to reach an obscuration between 2 and 8. Mean diameter was expressed by volume correspondent sphere (volume-weighted mean diameter [*D*_(4,3)_]), and the polydispersity of the size distribution was expressed by the *SPAN* value, calculated by Equation (2)

(2) $$SPAN=\frac{D\left(0.9\right)-D\left(0.1\right)}{D\left(0.5\right)}$$

where *D*_(0.9)_, *D*_(0.1)_ and *D*_(0.5)_ are the diameters at 90%, 10% and 50% under the cumulative size distribution curve by volume of particles, respectively. The specific surface (m^2^ g^−1^) was also determined for all samples considering particle density 1 g cm^−3^.

#### 2.5.2. Dynamic Light Scattering

Hydrodynamic mean diameter (z-average diameter) was determined by dynamic light scattering (DLS) using a ZetaSizer^®^ ZS instrument (Malvern Instruments, Malvern, UK) at 25 °C, after diluting the samples 500 times (*v*/*v*) in previously filtered (0.45 µm) MilliQ^®^ water.

#### 2.5.3. Nanoparticle Tracking Analysis

The Nanoparticle Tracking Analysis (NTA) was performed using a NanoSight^®^ LM20 instrument (NanoSight^®^, Salisbury, UK) at room temperature. Each sample was diluted 10,000 times (*v*/*v*) in MilliQ^®^ water, at a concentration suitable for NTA measurements, i.e., between 10^7^ and 10^9^ particles per mL, and injected in the sample chamber, until it was completely filled. A light beam laser at 640 nm passes through the chamber and the light individually scattered by the nanoparticles was captured at an angle of 90° by a CCD camera for 60 s with manual shutter and gain adjustments. The video showing the Brownian motion of the nanoparticles was analyzed by NTA 2.0 Build 127 software, correlating the movement to the diffusion coefficient, which is related to a determined particle size. The size distribution curves were expressed by number of particles, corresponding to the arithmetic values calculated by all particle sizes analyzed by the instrument. The NTA software is able to identify and track individual nanoparticles circulating in Brownian movement and relates the movement to the particle size according to the Stokes–Einstein equation, in which are considered the Boltzmann constant, temperature, medium viscosity, time and hydrodynamic radius.

### 2.6. Zeta Potential

Zeta potential was determined by ZetaSizer^®^ ZS (Malvern, UK), which physico-chemical principle is based on the electrophoretic mobility of colloidal systems. Before the analysis, each formulation was diluted 500 times in a 10 mmol L^−1^ sodium chloride aqueous solution, previously filtered (0.45 µm).

### 2.7. Potentiometry

The pH value for each formulation was determined in a DM-22 potentiometer (Digimed, Brazil) at 25 °C, previously calibrated with buffer solutions pH 4.0 and 7.0. The electrode was immersed directly in the formulation after preparation. The analysis was performed in triplicate batches.

### 2.8. Particle Size Stability in In Vitro Gastrointestinal Simulated Fluids

The simulated gastric fluid (SGF) (pH 1.2 and pepsin) and simulated intestinal fluid (SIF) (pH 7.5 and pancreatin) were used as media for the in vitro particle size stability study [36]. One milliliter of Cap(0.5)-Zn(25)-MLNC-Fur(0.45) was added to 9.0 mL of each medium and kept under magnetic stirring at 37 °C. Samples were collected just after adding the formulation, and after 1, 2 and 3 h of incubation [37]. The withdrawn samples were analyzed by laser diffraction (Malvern^®^ 2000 Mastersizer^®^, Malvern Instruments, Malvern, UK). The media were also analyzed to determine their particle distribution profiles.

### 2.9. In Vivo Studies

#### 2.9.1. Animals

All animal experimentations were performed in compliance Brazilian current legislation (Lei Arouca, Cobea, 2009) and the rules of the Veterinary Medical Ethics Code (Brazil, 2002). The protocols were approved by the Ethical Committee for the Animal Care and Use of the Instituto de Cardiologia, Brazil (#UP5037/14).

Male adult spontaneously hypertensive rats (SHR), 60 days old and weighting 200 ± 30 g were purchased from the Instituto de Cardiologia (Porto Alegre, Brazil) and allowed to acclimatize for at least two weeks before all experiments. At the beginning of the experiments, the animals presented a systolic pressure over 150 mmHg. The animals were housed in groups of four or five animals per cage (49 × 34 × 16 cm) under standard environmental conditions, controlled temperature (22 ± 2 °C), 12 h/12 h dark/light cycle, relative humidity of 50 ± 10%, and free access to standard diet and tap water.

#### 2.9.2. Treatment

The male adult spontaneously hypertensive rats were randomly divided into seven groups (*n* = 6 animals each). The treatment was administered by oral gavage: (1) control (poly(ethyleneglycol:water) (3:2; *v*/*v*); (2) captopril aqueous solution (0.5 mg mL^−1^); (3) captopril (0.5 mg mL^−1^) and furosemide (0.45 mg mL^−1^) in poly(ethyleneglycol):water (3:2; *v*/*v*) solution; (4) the formulation [Capt(0.5)-MLNC-Fur(0.45)] prepared without Zn^2+^; (6) the test formulations Capt(0.5)-Zn(25)-MLNC and Capt(0.5)-Zn(25)-MLNC-Fur(0.45). The oral treatments consisted of a furosemide dose of 3.6 mg kg^−1^ and a captopril dose of 4.0 mg kg^−1^ each 24 h for 5 days.

#### 2.9.3. Blood Pressure Measurements

Blood pressure was measured by tail-cuff plethysmography (V.2.11—Insight^®^), which is a non-invasive method. A cuff is placed in animal tail, inflated and a transducer registers the arterial pulse. From a scale of previous calibration, it is possible to determine the value for the first captured pulse and this value is considered the value of systolic blood pressure. When the pressure is slowly released, the blood passes through the cuff swelling the tail. The diastolic blood pressure is considered when the tail stops swelling and this measurement seems to be somewhat less reliable by indirect methods. The animals were preconditioned to the experimental conditions (heat and plethysmography) for five days to minimize measurement errors. Prior to the experiments, animals were previously kept at 40 °C in an acrylic containment box for 15 min, to make the pulsations of the tail artery detectable. In the first day of treatments, the pressure was measured at 0, 1, 2, 4, 6, 8, 10, 12, 14, 16, 18, 20, 22 and 24 h following oral administration and 0, 1, 2, 4, 6, 8, 10, 12 and 24 h on the fifth day. Results were expressed as percentage variations (%) relative to the basal pressure of each animal.

#### 2.9.4. Toxicological Evaluation

On the fifth day, the animals were kept under isoflurane anesthesia and the blood was collected from vena cava into a sodium EDTA tube for plasma, and a tube without anticoagulant for serum. The blood samples were centrifuged at 1500× *g* for 10 min and the supernatant was removed and stored at −80 °C until analysis. After the rats were euthanized, the brain, heart, spleen, kidneys, liver and lungs were collected, washed with a saline solution and observed for macroscopic morphological alterations.

##### Biochemical Analyses

Alanine aminotransferase (ALT), aspartate aminotransferase (AST), uric acid (UA), urea and creatinine (Cr) were analyzed in serum using commercial kits (ADVIA 1800 Siemens Healthcare Diagnostics Inc., Tarrytown, NY, USA). Sodium, chlorine and potassium were determined by ion selective electrode ADVIA 1800 (Siemens Healthcare Diagnostics Inc., Tarrytown, NY, USA).

##### Early Kidney Damage Markers

On the fifth day, after 12 h of the administration, the animals were housed individually for 12 h in metabolic cages with controlled food and water, in accordance with the Canadian Council on Animal Care. The urine samples were collected and kept refrigerated until the analysis. The pH and the specific gravity of the urine were analyzed using urine dipstick (Multistix 10 SG Siemens, Bayer Diagnostics, Tarrytown, NY, USA). Urinary creatinine levels (Cr-U) were evaluated by a commercial laboratory kit (Doles Reagents, Goiânia, Brazil). *N*-acetyl-beta-d-glucosaminidase (NAG) activity was verified by a spectrophotometer method (UV–VIS Hitachi spectrophotometer model U-1800^®^, Chiyoda, Tokyo, Japan) according to Horak and co-workers [38]. Microalbumin was analyzed by an immune turbidimetric method (ADVIA 1800 Siemens Healthcare Diagnostics Inc., Tarrytown, NY, USA).

##### Oxidative Status Evaluation

Lipid peroxidation was evaluated by quantifying malondialdehyde (MDA) in plasma samples. MDA levels were determined by a HPLC with a visible detector (WellChrom HPLC-VIS, Knauer, Germany), as described by Grotto and co-workers [39]. Before the injection of the samples into the HPLC, a hydrolysis step using 3 mol L^−1^ NaOH aqueous solution at 60 °C for 30 min was performed, followed by deproteinization and extraction with *n*-butanol.

Non-protein thiols were measured in the erythrocytes after centrifugation following the protocol developed by Ellman [40] with modifications. Briefly, aliquots (0.3 mL) of erythrocytes were added to a phosphate buffer 0.3 mol L^−1^ (0.85 mL), pH 7.4 and the reaction was analyzed in spectrophotometer (Biospectro, Brazil) at 412 nm after adding 10 mmol L^−1^ 5-5′-dithio-*bis*(2-nitrobenzoic) acid (DTNB) (0.05 mL).

### 2.10. Morphological Analysis and Kinetic Stability of the Selected Formulation

#### 2.10.1. Transmission Electron Microscopy (TEM)

The morphological analysis of the formulations was performed by transmission electron microscopy (TEM) (JEOL JEM 1200 Exll, Tokyo, Japan) at three different magnifications (50 K, 100 K and 300 K) in the Electron Microscopy and Microanalysis Center (CMM) at the Federal University of Rio Grande do Sul. Each formulation was diluted in a proportion of 1:10 in MilliQ^®^ water (*v*/*v*) and placed on a copper grid (400 mesh) coated with formvar-carbon (Electron Microscopy Sciences, Hatfield, PA, USA) and stained with 2% uranyl acetate aqueous solution. The samples were kept in a desiccator under vacuum until the analysis was performed at 80 kV.

#### 2.10.2. Atomic Force Microscopy (AFM)

Atomic Force Microscopy was performed in a NanoscopeIIIa Multimode, (Digital Instruments, Santa Barbara, CA, USA). For height (flat and three dimensional) and phase analysis, the software Nanoscope v5.31r1 (Bruker, Santa Barbara, CA, USA) was used. A small amount of sample was collected with a glass capillary and deposited on mica, previously fixed in the sample port with the aid of an adhesive double-sided tape. The sample was left to completely dry and, then, it was analyzed. To read the surface areas in AFM, tapping mode was employed for an analysis of 1 × 1 μm^2^ area. Each area was scanned with 256 points at 0.5003 Hz speed and range (*z*-axis) of 50 nm. The measurements were performed in air and room temperature (23 °C).

#### 2.10.3. Multiple Light Scattering Analysis

The kinetic stability of the system [Capt(0.5)-Zn(25)-MLNC-Fur(0.45)] was evaluated by multiple light scattering using a Turbiscan LAb^®^ instrument (Formulaction Co., Toulouse, France). The sample (20.0 mL) was placed in a cylindrical optical glass cell and inserted in the equipment without any dilution. The analysis was performed at 25 °C. The scanning was made every 40 µm, from the bottom to the top of the optical cell (55 mm), for 1 h every 5 min by a near infrared light source (λ = 880 nm). The instrument is equipped with two detectors that receive the transmitted and backscattered light at 0° and 135°, respectively.

### 2.11. Statistical Analysis

The statistical analyses were performed by using SPSS 18.0 for Windows and GraphPad Prism 5 Project software systems (La Jolla, CA, USA). The data were expressed as mean ± standard deviations (SD) or standard error of the mean (SEM). The level of significance was taken as *p* < 0.05 or *p* < 0.01 and it was determined using one-way analysis of variance (ANOVA) or repeated measures ANOVA followed by Bonferroni post hoc tests for multiple comparisons.

## 3. Results and Discussion

### 3.1. Development of Formulations

Captopril-surface functionalized MLNC and captopril-surface functionalized furosemide-loaded MLNC were developed in four steps: (a) self-assembling of the materials, (b) chitosan coating, (c) Zn^2+^ interfacial reaction and (d) complexation forming the ligand-Zn^2+^ organometallic complex on the nanocapsule surface. The first step of the synthesis was to produce the lipid-core nanocapsules [LNC or LNC-Fur(0.5)] using the solvent displacement method by self-assembling the materials in an aqueous medium. Then, the formulations were coated with 1% chitosan in 1% acetic acid aqueous solution. A higher amount of chitosan was used to coat those formulations compared to the concentration (0.3%) used in our previous study [27], as a higher amount of oily phase was used in the present work leading to a higher surface area to be coated.

**Figure 1:**
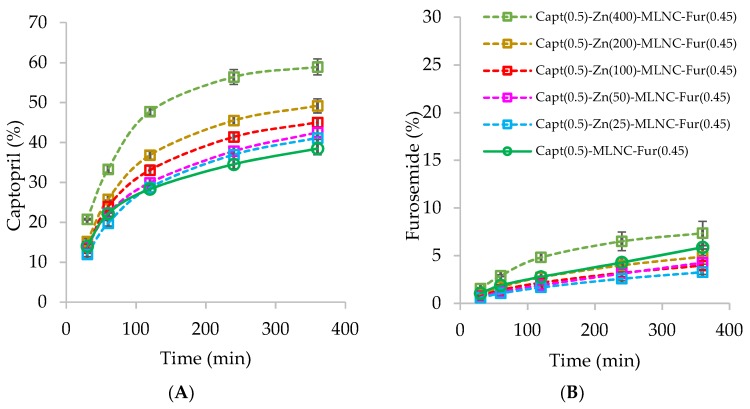
Dialysis in water for 6 h of formulations: Capt(0.5)-MLNC-Fur(0.45) prepared without Zn^2+^ and Capt(0.5)-Zn-MLNC-Fur(0.45) prepared using increased Zn^2+^ concentrations (25, 50, 100, 200 and 400 µg mL^−1^). **A**: dialysis of captopril and **B**: dialysis of furosemide. Legend for both graphics is shown in B.

In this way, it was necessary to select the optimal concentration of Zn^2+^ to form a coordination complex with chitosan and captopril on the nanocapsules surface. For this reason, different concentrations of Zn^2+^ were tested and the release of captopril was evaluated looking for the most sustained release profile. Six formulations of Capt(0.5)-Zn-MLNC-Fur(0.45) were prepared varying the concentrations of Zn^2+^ (0, 25, 50, 100, 200 and 400 µg mL^−1^) and maintaining constant the captopril concentration (0.5 mg mL^−1^). After 6 h, Capt(0.5)-Zn(400)-MLNC-Fur(0.45) released higher amount (*p* < 0.05) of captopril dialyzed (59%) than the other formulations (38% to 45%) (Figure 1A). The results indicated that captopril at 0.5 mg mL^−1^ in some degree (40% to 50%) was bound to the nanocapsule surface when Zn^2+^ was used at a concentration from 25 to 200 µg mL^−1^. Additionally, the results demonstrated that the release of captopril was also sustained from Capt(0.5)-MLNC-Fur(0.45), in the absence of Zn^2+^, since the concentration of drug released was 38%, similar to that released from the formulation containing 25 µg mL^−1^ of Zn^2+^. This result can be explained by an electrostatic interaction of the drug with chitosan on the nanocapsule surface. Furthermore, Capt(0.5)-MLNC-Fur(0.45), Capt(0.5)-Zn(25)-MLNC-Fur(0.45) and Capt(0.5)-Zn(50)-MLNC-Fur(0.45) released lower amounts of captopril than Capt(0.5)-Zn(100)-MLNC-Fur(0.45) and Capt(0.5)-Zn(200)-MLNC-Fur(0.45) (*p* < 0.05). Comparing the formulation prepared using 25 µg mL^−1^ of Zn^2+^ with those prepared without zinc or using 50 µg mL^−1^ of Zn^2+^, no statistical difference was observed in all time-periods analyzed (*p* > 0.05). However, formulations prepared without zinc or using 50 µg mL^−1^ of Zn^2+^ were different from each other from 4 to 6 h (*p* < 0.05). These results indicated higher captopril diffusion by increasing the concentration of Zn^2+^, probably due to the metal ion oversaturated concentration in relation to the chitosan available on the nanocapsules surface to form the organometallic complex. The excess of Zn^2+^ reacts with captopril in the aqueous phase, which salt can freely diffuse through the dialysis membrane.

Capt(0.5)-Zn(25)-MLNC-Fur(0.45) presented pH of 4.05 ± 0.02, which was the value used to calculate the lipophilicity of furosemide. Furosemide has a log D of 1.91 at pH 4.0. Considering our previous study [41], this value suggested that furosemide is likely distributed at the lipid-core and at the polymer wall of the nanocapsules. In this way, if the integrity of the nanocapsules is maintained, the furosemide might present a slow and sustained release profile. Indeed, the furosemide dialysis showed less than 8% of furosemide released within 6 h of experiment for all formulations tested (Figure 1B). Capt(0.5)-Zn(25)-MLNC-Fur(0.45) and Capt(0.5)-Zn(400)-MLNC-Fur(0.45) released 3.3 ± 0.3% and 7.4 ± 1.5% of furosemide after 6 h of experiment and the furosemide dissolution in 6 h from Capt(0.5)-MLNC-Fur(0.45) was 5.9 ± 0.2%. Slow release of furosemide from PLGA and PCL-PEG nanoparticles has already been described [42]. The authors showed that the higher the proportion of polymers in the formulation, the slower the drug release. In our formulations, the ratio polymer/drug was at least 3.5-times higher than the ratio used by Youm and co-workers [42] explaining the slow release observed for our formulations.

Hence, the results demonstrated that the optimal concentration of Zn^2+^ to react with chitosan and captopril, in our experimental conditions, is 25 µg mL^−1^. Therefore, we selected the Capt(0.5)-Zn(25)-MLNC-Fur(0.45) formulation for the subsequent steps of the study.

After optimizing the concentration of Zn^2+^, the efficiency of the organometallic complex formation was evaluated by ultrafiltration/centrifugation. Firstly, we determined that captopril was recovered in 92% in the ultrafiltrate when an aqueous solution of captopril, Zn^2+^ and polysorbate 80 was assayed. Furthermore, captopril was retained in 35.5% by the membrane of the ultrafiltration unit after assaying an aqueous solution of captopril and chitosan. For Capt(0.5)-Zn(25)-MLNC and Capt(0.5)-Zn(25)-MLNC-Fur(0.45), captopril was retained in 37.69% and 39.91%, respectively, while for Capt(0.5)-MLNC-Fur(0.45)] it was retained in 36.62%.The results indicated that captopril could bind to the surface of the nanocapsules by electrostatic interaction with chitosan besides the coordination bond corroborating the results described above. The furosemide encapsulation efficiency was 97.47%, a value consistent with the data obtained by dialysis technique, which showed 3.3% of the drug released in 6 h of experiment.

### 3.2. Physico-Chemical Characteristics of the Selected Formulations

Capt(0.5)-Zn(25)-MLNC and Capt(0.5)-Zn(25)-MLNC-Fur(0.45) were analyzed by laser diffraction to determine the particle size distribution in a range between 20 nm and 2 mm. After discarding the presence of microparticles or microaggregates in the formulations, the samples were analyzed by DLS that is a more suitable technique to characterize particles in the nanometric range [43]. Laser diffraction data were plot as radar charts to characterize the fingerprints of the formulations [44]. Capt(0.5)-Zn(25)-MLNC and Capt(0.5)-Zn(25)-MLNC-Fur(0.45) showed radar chart shapes coherent to unimodal size distributions (Figure S1). Sauter mean diameters, *D* [3,2], were respectively 114 ± 1 nm and 115 ± 3 nm, and volume weighted mean diameters, *D* [3,4], were respectively 158 ± 1 nm and 142 ± 4 nm. The polydispersity, described as SPAN, were 1.408 ± 0.050 and 1.250 ± 0.113 and the specific surface areas were 52.5 ± 0.7and 52.1 ± 1.5 m^2^ g^−1^, respectively. Furthermore, the median diameters based on the number of particles were 64 ± 1 and 66 ± 3 nm, respectively. Regarding the DLS data, Capt(0.5)-Zn(25)-MLNC and Capt(0.5)-Zn(25)-MLNC-Fur(0.45) showed unimodal size distributions with z-average diameters of 173 ± 8 nm and 153 ± 6 nm with PDI values of 0.22 ± 0.02 and 0.18 ± 0.03, respectively. Considering the zeta potential analysis, the formulations had +18.1 ± 1.3 and +12.2 ± 1.1 mV, respectively, evidencing the positively charged particle surface by the chitosan coating.

The formulations were also analyzed by NTA. Compared to DLS, NTA technique has the advantage of considering the individual particles, as it has a digital camera that allows recording the scattered light from the dispersed nanoparticles in water, furnishing a better resolution for multimodal samples and enabling a direct visual information of aggregation phenomena [45]. The results showed unimodal size distribution profiles for both formulations, which had median diameters of 177 ± 2 nm and 165 ± 6 nm, mean hydrodynamic diameters of 206 ± 2 nm and 168 ± 5 nm with polydispersity values of 1.35 ± 0.07 and 0.77 ± 0.08, respectively. NTA also furnished the particle number density of the formulations that were 3.85 ± 0.28 × 10^13^ and 0.82 ± 0.14 × 10^13^ nanocapsules per mL, respectively.

### 3.3. Particle Size Stability in Gastrointestinal Simulated Fluids

Capt(0.5)-Zn(25)-MLNC and Capt(0.5)-Zn(25)-MLNC-Fur(0.45) were designed as liquid dosage forms for oral administration to SHR. In this way, before performing the in vivo biological evaluation, we carried out a particle size stability study in simulated gastric or intestinal fluids using Capt(0.5)-Zn(25)-MLNC-Fur(0.45) as test formulation.

The simulated fluids analyzed by laser diffraction did not show the presence of particles in the range from 20 nm to 2 mm. After adding Capt(0.5)-Zn(25)-MLNC-Fur(0.45) in the SGF, the radar chart shapes after 1, 2 and 3 h were overlapped to that of the beginning of the experiment (Figure S2A). Nevertheless, when the formulation was added into SIF, changes in the radar chart shapes were observed as a function of time (Figure S2B). The *D* [3,4] *v* value increased from 177 to 332 nm after 1 h remaining constant until 3 h, and *d*(0.9) *v* value increased from 311 to 454 nm after 1 h and to 688 nm after 2 h remaining constant until 3 h.

Different kinds of nanoparticles systems had already been tested for stability in simulated media. Insulin-chitosan nanoparticles which had had their absorbance evaluated until 7 h in gastric and intestinal simulated media showing that nanoparticles immediately dissolved in gastric medium and, after 7 h, 84% were dissolved in intestinal medium, while in water, there was no change in optical density [46]. Roger, Lagarce and Benoit performed a stability study of lipid nanoparticles in fasted state (FaSSIF) and in fed state (FeSSIF) fluids demonstrating that the size of the nanoparticles was not modified after 3 h [47]. Lipid nanoparticles were also studied in 1% (*w*/*v*) in simulated intestinal (until 5 h) and gastric media (until 3 h) and size distribution was evaluated by PCS while triglyceride degradation was assessed by a colorimetric assay. It was observed that non-coated lipid nanoparticles present a great aggregation in gastric medium being necessary to perform a protection with coatings such as poloxamers or PEG-stearate. It was also observed the process of aggregation in intestinal medium for non-coated particles and triglyceride degradation, that was not observed with coated nanoparticles [48]. Zimmerman and Müller studied the stability of solid lipid nanoparticles with different lipids and surfactants/stabilizers in gastrointestinal simulated media and it was observed that the influence of the low pH was higher than the electrolyte concentration. Particles developed using Tween 80, which is present in our systems, showed better particle stability probably due to a steric stabilization. Although formulations can present particle aggregation in simulated media in vitro, it has to be considered that the influence of ionic strength and pH are the unique parameters controlled, while experiments carried out in vivo represent much more complex systems, in which the presence of proteins and enzymatic degradation could also lead to deaggregation of the systems [49]. Based on that, we considered that the observed variation in particle size in simulated intestinal medium is acceptable to follow up the in vivo experiments, which may appropriately provide information on the potential of the formulation for oral administration.

### 3.4. In Vivo studies

As previous results showed that the formulations presented a unimodal distribution, a sustained release profile and an acceptable stability in simulated media, they were able to be tested in in vivo studies to compare their effect and toxicity to the solution of both drugs.

#### 3.4.1. Blood Pressure Measurements

The study was conducted to compare Capt(0.5)-Zn(25)-MLNC with the formulation containing the association captopril and furosemide [Capt(0.5)-Zn(25)-MLNC-Fur(0.45)]. Considering that captopril is interacting on the nanocapsule surface with both Zn^2+^ (organometalic complex) and chitosan (electrostatic interactions), as mentioned above, we also studied Capt(0.5)-MLNC-Fur(0.45), prepared without Zn^2+^. Furthermore, captopril solution and captopril + furosemide solution were used to determine the effect of the nanoencapsulation of the drugs on the blood pressure.

**Figure 2:**
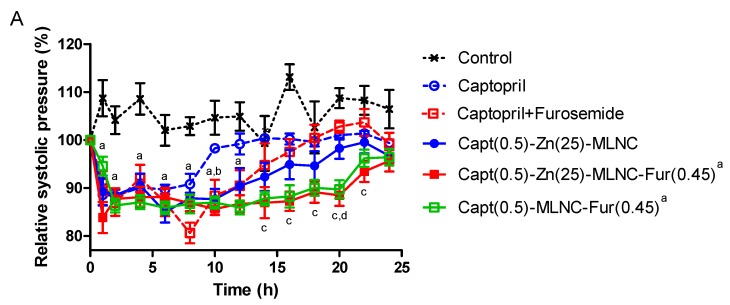

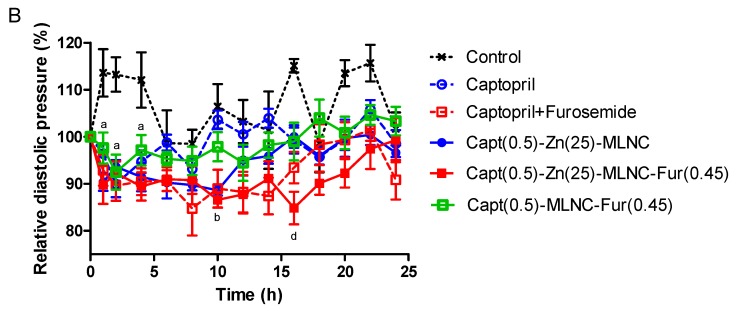
Systolic blood pressure (**A**) and diastolic blood pressure (**B**) (% from the basal pressure) from the first day of administration of solutions or formulations (8 mL kg^−1^) by indirect tail method (*n* = 6). Data are represented as means ± standard error of the mean. ^a^ Significantly different from control; ^b^ significantly different from captopril; ^c^ significantly different from captopril + furosemide; ^d^ significantly different from Capt(0.5)-Zn(25)-MLNC (two-way ANOVA/Bonferroni; *p* < 0.05).

The mean basal systolic blood pressure values of SHR were over 180 mmHg. In the first day of experiment, the captopril aqueous solution kept the systolic pressure down in relation to the control group for 8 h (*p* < 0.05), while the Capt(0.5)-Zn(25)-MLNC formulation prolonged this effect until 10 h (*p* < 0.05). A similar profile was observed for the captopril + furosemide solution (*p* < 0.01). Interestingly, the antihypertensive effect was prolonged to 20 h in the Capt(0.5)-MLNC-Fur(0.45) and 22 h in the Capt(0.5)-Zn(25)-MLNC-Fur(0.45) treated animals (*p* < 0.05) compared to control. Moreover, Capt(0.5)-MLNC-Fur(0.45) and Capt(0.5)-Zn(25)-MLNC-Fur(0.45) were more efficient than either the captopril solution from 14 to 20 and 22 h (*p* < 0.05), respectively; as well as from the captopril + furosemide solution (*p* < 0.05) (Figure 2A).

In the diastolic pressure, the captopril and the captopril + furosemide solutions, as well as the Capt(0.5)-Zn(25)-MLNC and Capt(0.5)-MLNC-Fur(0.45) formulations kept the diastolic pressure down until 4 h of experiment, compared to control group (*p* < 0.05) on the first day of experiments. The Capt(0.5)-Zn(25)-MLNC-Fur(0.45) group was the only that kept the diastolic pressure down until 16 h (*p* < 0.05). Capt(0.5)-Zn(25)-MLNC-Fur(0.45), compared to captopril solution, showed a more pronounced antihypertensive effect at 10 h (*p* < 0.05). Therefore, Capt(0.5)-Zn(25)-MLNC-Fur(0.45) presented a better time effect profile compared to other groups, demonstrating that the formulation promoted a longer antihypertensive effect.

On the fifth day, a decrease in time effect of systolic blood pressure comparing to the first day was observed for all groups, except for Capt(0.5)-Zn(25)-MLNC-Fur(0,45). In relation to the control group, the captopril solution reduced the time effect to 4 h (*p* < 0.05), while the Capt(0.5)-Zn(25)-MLNC reduced 10 h (*p* < 0.05). In addition, the captopril + furosemide solution showed antihypertensive effect until 6 h (*p* < 0.05), a shorter time effect compared to the Capt(0.5)-Zn(25)-MLNC formulation, which maintained the antihypertensive effect until 24 h, compared to the control group (*p* < 0.05). This effect was significantly more effective than those observed for captopril + furosemide and Capt(0.5)-Zn(25)-MLNC formulation (*p* < 0.05). Capt(0.5)-MLNC-Fur(0.45) did not maintain the antihypertensive effect for 24 h, while Capt(0.5)-Zn(25)-MLNC-Fur(0.45) showed the longest antihypertensive effect for systolic pressure (24 h) compared to all groups (*p* < 0.05).

**Figure 3:**
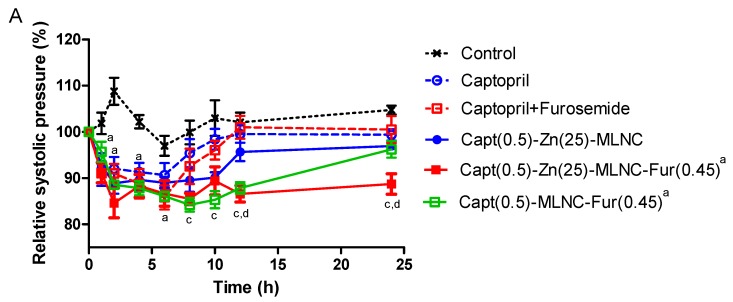

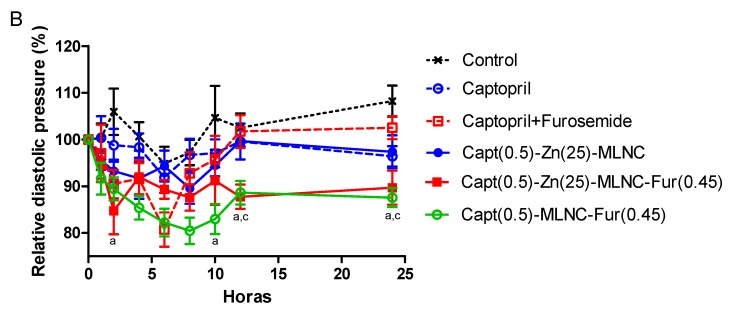
Systolic blood pressure (**A**) and diastolic blood pressure (**B**) (% from the basal pressure) from the fifth day of administration of solutions or formulations (8 mL kg^−1^) by indirect tail method (*n* = 6). Data are represented as means ± standard error of the mean. ^a^ Significantly different from control; ^b^ significantly different from captopril; ^c^ significantly different from captopril + furosemide; ^d^ significantly different from Capt(0.5)-Zn(25)-MLNC (two-way ANOVA/Bonferroni; *p* < 0.05).

Evaluating the diastolic pressure on the fifth day (Figure 3B), no difference was observed when captopril solution, Capt(0.5)-Zn(25)-MLNC and captopril + furosemide solution were compared to the control group (*p* < 0.05). On the other hand, Capt(0.5)-Zn(25)-MLNC-Fur(0.45) was significantly different from control group between 10 and 24 h (*p* < 0.05) and from captopril + furosemide solution at 12 and 24 h (*p* < 0.05). Capt(0.5)-Zn(25)-MLNC-Fur(0.45) and Capt(0.5)-MLNC-Fur(0.45) presented an antihypertensive effect until 24 h (*p* < 0.05).

In the present study, our strategy was based on the surface functionalization by forming an organometallic complex using Zn^2+^ binding captopril to the chitosan-coated lipid-core nanocapsules and loading furosemide within the lipophilic inner phase of those multi-wall nanocapsules. Capt(0.5)-Zn(25)-MLNC-Fur(0.45) orally administered showed the most favorable results of antihypertensive effect. Improvements in the intensity and duration of the pharmacodynamic effect compared to the result of captopril + furosemide solution highlighted that the supramolecular structure was physico-chemically stable in the gastrointestinal tract, crossed the intestinal barrier and reached the blood stream. These results indicates that multi-wall lipid-core nanocapsule acted as an in vivo controlled release system for both drugs capable of increasing the pharmacological effect as well.

The antihypertensive effect of nanoparticles in SHR through plethysmographic method after oral administration has been studied previously. Antal and co-workers [50] evaluated the antihypertensive effect of 25 mg/kg per day aliskiren-loaded magnetic poly(d,l-lactide) nanoparticles (NP) after three weeks of treatment in SHR. The blood pressure was evaluated every week and the results showed that the blood pressure of aliskiren treated group was lower than the control group. Besides, the aliskiren-NP group decreased in blood pressure compared to the aliskiren and the control groups. The study evaluated a longer time of administration, however variations of pressure along the day were not observed, but it was proved that the nanoparticles could protect the drug from degradation and improve its bioavailability. A similar study was performed evaluating systolic arterial blood pressure 0, 0.25, 0.5, 1, 2, 4, 7 and 10 h following oral administration of nifedine (NFP)-loaded polymeric nanocapsules, prepared with PCL, polylactic and glycolic acid (1:1) copolymers (PLAGA), or Eudragit RL/RS (Eudragit) [51]. The oral administration of NFP in PEG solution promoted a fall in systolic arterial blood pressure in 0.25 h that returned in 10 h, while PCL and PLAGA nanoparticles significantly reduced the systolic arterial blood pressure and maintained the hypotensive effect up to 10 h. However, Eudragit nanoparticles had a progressive antihypertensive effect until its maximal effect in 2 h that was not different from 10 h. Similar results were obtained in the present work, where the nanocapsules promoted a more extended antihypertensive effect compared to drugs in PEG solution, however, in the present study, we could deliver two drugs (captopril on the surface and furosemide in the core) in the same structure, which was not described previously in the literature for hypertension, improving the antihypertensive effect when compared to the formulation containing only captopril.

#### 3.4.2. Toxicological Evaluation

##### Metabolic Cages

In spite of no alterations in food consumption, there was a significant decrease (*p* < 0.05) in the water consumption in the Capt(0.5)-Zn(25)-MLNC-Fur(0.45) treated animals compared to Capt(0.5)-Zn(25)-MLNC group (Table 1). The percentage of urine collected after 12 h of the last administration in metabolic cages was significantly (*p* < 0.05) increased in the Capt(0.5)-Zn(25)-MLNC group compared to control and captopril treated groups (Table 1). The relation between urine and water better represents the water balance and its impact in blood pressure. No alteration in this relation was noted after treatments.

##### Biochemical Analyses

After 24 h of the fifth day of administration there were no observed significant (*p* > 0.05) alterations in serum alanine aminotransferase (ALT), aspartate aminotransferase (AST), urea, creatinine, and uric acid among the groups (Table 2), showing no potential hepatotoxicity or nephrotoxicity of the nanoformulation after 5 days of administration. The analysis of the ions in serum also showed no significant alterations in sodium, potassium or chloride (Table 2; *p* > 0.05). In a previous study using isolated perfused kidneys from SHRs, it was revealed an intrinsic renal abnormality in sodium excretion that may contribute to the maintenance of hypertension in SHR [52].

##### Early Kidney Damage Markers

**Figure 4:**
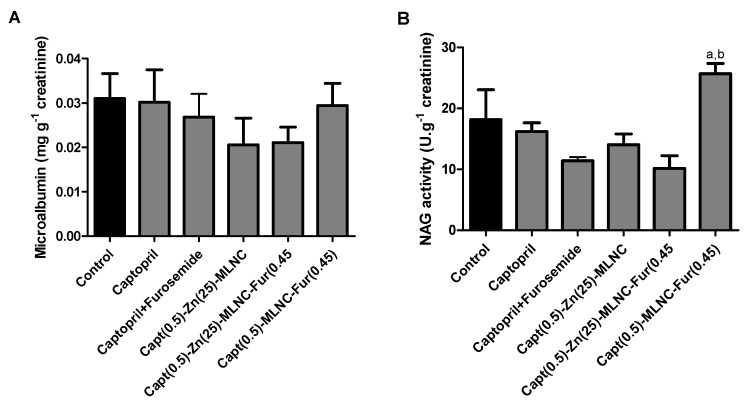
Microalbumin (mg g^−1^ creatinine) (**A**) and *N*-acetyl-β-d-glucosaminidase (NAG) activity (U g^−1^ creatinine) (**B**) in urine of spontaneously hypertensive rats (SHR) after 5 days of oral treatment (8 mL kg^−1^) with solutions or formulations (*n* = 6). Values are expressed as mean ± SEM. Data were analyzed by ANOVA/Bonferroni. ^a^ Significantly different from Captopril + Furosemide (*p* < 0.05); ^b^ significantly different from Capt(0.5)-Zn(25)-MLNC-Fur(0.45).

For all the groups the pH values of urine ranged between 7.0–7.3 and the specific gravity between 1.01 and 1.03. The tests for glucose, bilirubin, ketone bodies, nitrites and leucocytes were negative in urine for all groups. No alterations were observed in microalbumin excretion (Figure 4A), however, a significant (*p* < 0.05) increase in urinary NAG activity was noted in Capt(0.5)-MLNC-Fur(0.45) group compared to Captopril + Furosemide and Capt(0.5)-Zn(25)-MLNC-Fur(0.45) treated animals (Figure 4B). Hypertension is an established consequence of chronic renal disease and is often observed in patients with focal segmental glomerulosclerosis and with membranoproliferative glomerulonephritis [53]. Therefore, it might be assumed that the formulation Capt(0.5)-Zn(25)-MLNC-Fur(0.45) has a potential to protect the kidneys of SHR from the increased hypertension that the formulation Capt(0.5)-MLNC-Fur(0.45) has not.

##### Macroscopical Organs Analyses

The relative mass of organs is presented in Table 3; no significant alterations were noted in liver, spleen, kidneys, lungs, and heart. Interestingly, a significant increase was observed (*p* < 0.05) in relative brain weight of Capt(0.5)-MLNC-Fur(0.45) treated animals compared to control and Capt(0.5)-Zn(25)-MLNC groups. It is well known that the SHR brain have a smaller volume compared to normotensive Wistar-Kyoto rats of the same age [54]. Animals treated for hypertension can have a neuroprotection to damage caused by it. As an example, a study using losartan administered orally from fourth to ninth weeks to animals in drinking water resulted in an increase of the relative brain weight, compared to the relative brain weight of the untreated SHR [55].

##### Oxidative Status Evaluation

**Figure 5:**
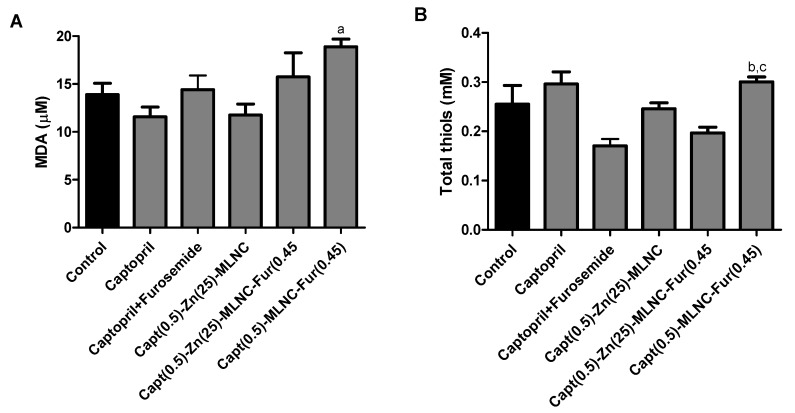
Malondialdehyde (MDA) (µM) (**A**) and total thiols (mM) (**B**) of spontaneously hypertensive rats (SHR) after 5 days of oral treatment (8 mL kg^−1^) with solutions or formulations (*n* = 6). Values are expressed as mean ± SEM. Data were analyzed by ANOVA/Bonferroni. ^a^ Significantly different from Capt(0.5)-Zn(25)-MLNC; ^b^ significantly different from Captopril + Furosemide; ^c^ significantly different from Capt(0.5)-Zn(25)-MLNC-Fur(0.45).

MDA and total thiols were analyzed in plasma and erythrocytes, respectively, as biomarkers of oxidative stress. Oxidative stress develops when the balance between pro-oxidants and antioxidants becomes uncontrolled and tips in favor of pro-oxidants. Oxidative stress is a common feature of hypertensive disorders from diverse origins and it is higher in SHR, compared to non-hypertensive ones [56]. Capt(0.5)-MLNC-Fur(0.45) significantly increased (*p* < 0.05) the MDA values compared to Capt(0.5)-Zn(25)-MLNC (Figure 5A). Additionally, in erythrocytes, a significant increase in total thiols in animals treated with Capt(0.5)-MLNC-Fur(0.45) was noted, compared to the Captopril + Furosemide and Capt(0.5)-Zn(25)-MLNC-Fur(0.45) treated groups (Figure 5B), this is known as a compensatory response of the organism. In this line, two aspects were evaluated: the self-effect of hypertension on oxidative stress (no alteration was observed in the present protocol) and the self-formulation effect in oxidative potential, that was observed in the Capt(0.5)-MLNC-Fur(0.45).

Analyzing all the results, an extended antihypertensive action could be observed when furosemide was associated with captopril in the formulation. Among them, Capt(0.5)-Zn(25)-MLNC-Fur(0.45) presented the best results, with more extended antihypertensive effect and not presenting any toxic damage. The results also showed that the presence of zinc in the formulation was relevant, since it presented a longer lasting effect compared to a similar formulation without the metal, which also caused toxic changes.

### 3.5. Morphological Analysis and Kinetic Stability of Capt(0.5)-Zn(25)-MLNC-Fur(0.45)

**Figure 6:**
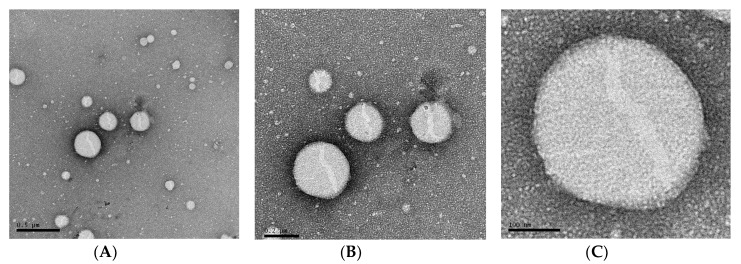
Morphological analysis by Transmission Electron Microscopy of Capt(0.5)-Zn(25)-MLNC-Fur(0.45), diluted in a proportion of 1:10 in MilliQ^®^ water (*v*/*v*), placed on a copper grid (400 mesh) coated with formvar-carbon (Electron Microscopy Sciences) and stained with 2% uranyl acetate aqueous solution. The samples were kept in a desiccator under vacuum until the analysis was performed at 80 kV [bars = 500 in (**A**), 200 in (**B**) and 100 nm in (**C**)].

**Figure 7:**
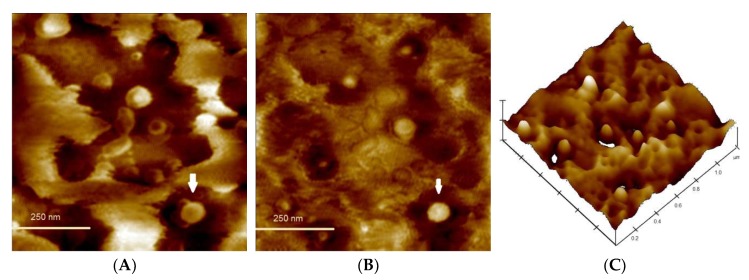
Phase (**A**) and height (**B**,**C**) 3D contrast images of Capt(0.5)-Zn(25)-MLNC-Fur(0.45). Sample was deposited on mica, left to completely dry and, then, analyzed by tapping mode of 1 × 1 μm^2^ area, which was scanned with 256 points at 0.5003 Hz speed and range (*z*-axis) of 50 nm, in air at room temperature (23 °C).

Transmission Electron Microscopy (TEM) analysis showed presence of spherical nanoparticles (Figure 6). The nanocapsules are surrounded by smaller structures (darker zone). The data obtained by Atomic Force Microscopy allowed observing the chitosan coating around the particle (arrow in Figure 7A,B) beyond confirming the nanometrical size and spherical shape of the particles. The 3D image demonstrated better the height perspective of the Capt(0.5)-Zn(25)-MLNC-Fur(0.45) particle and showed a smooth surface (Figure 7C) and a capsular structure.

The Capt(0.5)-Zn(25)-MLNC-Fur(0.45) formulation stability was evaluated by Multiple light scattering (Turbiscan^®^) during one hour at room temperature and an increased backscattering signal in the bottom and top of the cylindrical glass tube could be verified, indicating creaming and sedimentation phenomena which are considered reversible. The values were low indicating the nanoformulation is physically stable (Figure S3) and there was no variation in the middle of the glass tube. According to Celia and co-workers [57], only for values over 10%, positive or negative, could the system be considered unstable, thus, the formulation was considered physically stable as the values were under 2%.

## 4. Conclusions

Multi-wall lipid-core nanocapsules complexed with Zn^2+^ containing captopril bonded to the surface and furosemide encapsulated in the lipid core in the same structure were developed. For the first time, this nanotechnological approach was used to drug combination therapy as an alternative to hypertension treatment. According to the specific physico-chemical properties of each drug, captopril (able to coordinate to a metal ion) and furosemide (able to be encapsulated in the oily core), we could formulate both in the same nanostructure. These formulations, after oral administration, kept the antihypertensive effect of the drugs and also prolonged it compared to solutions, showing promising results for Capt(0.5)-Zn(25)-MLNC and also for Capt(0.5)-Zn(25)-MLNC-Fur(0.45). The association of furosemide into the nanocapsule lipid-core produced a more prolonged effect of the formulation compared to the drug solution and compared to the formulation containing only captopril. Even with the possibility of captopril to be bond to the surface by electrostatic attraction (without the metal ion), the results demonstrated that the presence of Zn^2+^ prolonged the antihypertensive effect of the formulation when Capt(0.5)-Zn(25)-MLNC-Fur(0.45) was compared to Capt(0.5)-MLNC-Fur(0.45). Furthermore, the toxicological studies in SHR proved that Capt(0.5)-MLNC-Fur(0.45) raised NAG activity, MDA and total thiols values, which was not observed for the same formulation containing Zn^2+^. Lastly, Capt(0.5)-Zn(25)-MLNC-Fur(0.45) proved to be an interesting strategy to associate drugs with different mechanisms to reach an antihypertensive effect, which formulation was developed in one-pot synthesis differently from most of the methods currently employed that are time-consuming and needs purification steps.

## Figures and Tables

**Table 1:** Relative water consumption, urine volume and urine/water relation of spontaneously hypertensive rats (SHR) after 5 days of oral treatment (8 mL kg^−1^) with solutions or formulations (*n* = 6). The animals were kept in metabolic cages for 12 h. Values are expressed as mean ± SEM. Data were analyzed by ANOVA /Bonferroni.

Group	Water Consumption (%)	Urine Volume (%)	Water/Urine Relation
Control	9.1 ± 0.5	1.8 ± 0.2	19.6 ± 2.1
Captopril	11.3 ± 0.9	1.9 ± 0.1	17.6 ± 1.5
Captopril + Furosemide	9.3 ± 0.7	2.2 ± 0.2	24.3 ± 1.8
Capt(0.5)-Zn(25)-MLNC	12.0 ± 0.7	3.3 ± 0.4 ^a,b^	27.7 ± 2.9
Capt(0.5)-Zn(25)-MLNC-Fur(0.45)	8.7 ± 0.5 ^c^	2.4 ± 0.2	28.3 ± 2.5
Capt(0.5)-MLNC-Fur(0.45)	10.5 ± 0.5	2.0 ± 0.2	18.9 ± 2.3

^a^ Significantly different from control. ^b^ Significantly different from captopril. ^c^ Significantly different from Capt(0.5)-Zn(25)-MLNC.

**Table 2:** Biochemical analyses in serum of spontaneously hypertensive rats (SHR) after 5 days of oral treatment (8 mL kg^−1^) with solutions or formulations (*n* = 6). Values are expressed as mean ± SEM. Data were analyzed by ANOVA.

Group	ALT (U L^−1^)	AST (U L^−1^)	Urea (mg dL^−1^)	Creatinine (mg dL^−1^)	Uric Acid (mg dL^−1^)	Na^+^ (mmol L^−1^)	K^+^ (mmol L^−1^)	Cl^−^ (mmol L^−1^)
Control	62.2 ± 7.0	141.3 ± 5.1	98.2 ± 3.2	4.4 ± 0.2	110.8 ± 15.5	0.25 ± 0.01	58.2 ± 0.8	0.8 ± 0.2
Captopril	60.7 ± 5.1	145.2 ± 2.8	102.7 ± 2.0	5.1 ± 0.3	111.8 ± 10.2	0.27 ± 0.02	60.7 ± 3.2	1.4 ± 0.1
Captopril + Furosemide	65.5 ± 4.8	137.4 ± 7.6	85.0 ± 9.6	4.5 ± 0.2	124.3 ± 20.6	0.22 ± 0.02	51.5 ± 2.6	1.2 ± 0.4
Capt(0.5)-Zn(25)-MLNC	55.8 ± 3.3	143.7 ± 1.9	101.8 ± 1.4	4.7 ± 0.2	105.5 ± 7.5	0.27 ± 0.02	53.5 ± 2.4	1.3 ± 0.2
Capt(0.5)-Zn(25)-MLNC-Fur(0.45)	76.8 ± 11.9	142.0 ± 1.1	99.2 ± 0.8	4.4 ± 0.1	125.0 ± 23.4	0.26 ± 0.01	60.2 ± 1.2	0.8 ± 0.1
Capt(0.5)-MLNC-Fur(0.45)	58.0 ± 3.9	143.4 ± 0.5	102.1 ± 9.3	4.1 ± 0.1	107.4 ± 8.1	0.25 ± 0.02	58.1 ± 3.9	1.1 ± 0.1

**Table 3:** Relative organs mass of spontaneously hypertensive rats (SHR) after 5 days of oral treatment (8 mL kg^−1^) with solutions or formulations (*n* = 6). Values are expressed as mean ± SEM. Data were analyzed by ANOVA/Bonferroni.

Group	Liver (%)	Spleen (%)	Right Kidney (%)	Left Kidney (%)	Lungs (%)	Heart (%)	Brain (%)
Control	4.4 ± 0.14	0.17 ± 0.003	0.40 ± 0.004	0.41 ± 0.012	0.51 ± 0.02	0.41 ± 0.009	0.78 ± 0.017
Captopril	4.8 ± 0.09	0.18 ± 0.002	0.39 ± 0.017	0.39 ± 0.015	0.63 ± 0.07	0.41 ± 0.007	0.81 ± 0.013
Captopril + Furosemide	4.3 ± 0.14	0.18 ± 0.004	0.41 ± 0.006	0.40 ± 0.014	0.53 ± 0.03	0.44 ± 0.004	0.81 ± 0.021
Capt(0.5)-Zn(25)-MLNC	4.6 ± 0.14	0.18 ± 0.003	0.38 ± 0.004	0.39 ± 0.007	0.52 ± 0.02	0.42 ± 0.008	0.86 ± 0.024
Capt(0.5)-Zn(25)-MLNC-Fur(0.45)	4.3 ± 0.12	0.17 ± 0.003	0.39 ± 0.006	0.39 ± 0.006	0.49 ± 0.02	0.43 ± 0.012	0.76 ± 0.023
Capt(0.5)-MLNC-Fur(0.45)	4.3 ± 0.05	0.18 ± 0.005	0.39 ± 0.006	0.39 ± 0.006	0.57 ± 0.03	0.41 ± 0.007	0.87 ± 0.009 ^a,b^

^a^ Significantly different from control. ^b^ Significantly different from Capt(0.5)-Zn(25)-MLNC-Fur(0.45).

## Supplementary Materials

The following are available online at https://www.mdpi.com/1999-4923/12/1/80/s1, Figure S1: Radar chart of laser diffraction data obtained by volume and by number of particles for Capt(0.5)-Zn(25)-MLNC and Capt(0.5)-Zn(25)-MLNC-Fur(0.45), Figure S2: Radar plots of laser diffraction data: A) Evolution of particle size distributions as a function of time (0, 1, 2 and 3 h) after adding Capt(0.5)-Zn(25)-MLNC-Fur(0.45) in simulated gastric fluid (SGF); (B) Evolution of particle size distributions as a function of time (0, 1, 2 and 3 h) after adding Capt(0.5)-Zn(25)-MLNC-Fur(0.45) in simulated intestinal fluid (SIF), Figure S3: Physical stability evaluation of Capt(0.5)-Zn(25)-MLNC-Fur(0.45) by Multiple Light Scattering.
