# Supplementary material for: Oral Treatment of Spontaneously Hypertensive Rats with Captopril-Surface Functionalized Furosemide-Loaded Multi-Wall Lipid-Core Nanocapsules
Source: Pharmaceutics. 2020 Jan 18;12(1):80. doi: 10.3390/pharmaceutics12010080 (PMC7022513; doi:10.3390/pharmaceutics12010080)
Supplement: Supplementary file 1 [file pharmaceutics-12-00080-s001.pdf]

pharmaceutics

Supplementary Materials: Oral Treatment of Spontaneously Hypertensive Rats with Captopril-Surface Functionalized Furosemide-Loaded Multi-Wall Lipid-Core Nanocapsules

Cecilia B. Michalowski, Marcelo D. Arbo, Louise Altknecht, Andréia N. Anciuti, Angélica S. G. Abreu, Luciana M. R. Alencar, Adriana R. Pohlmann, Solange C. Garcia and Sílvia S. Guterres

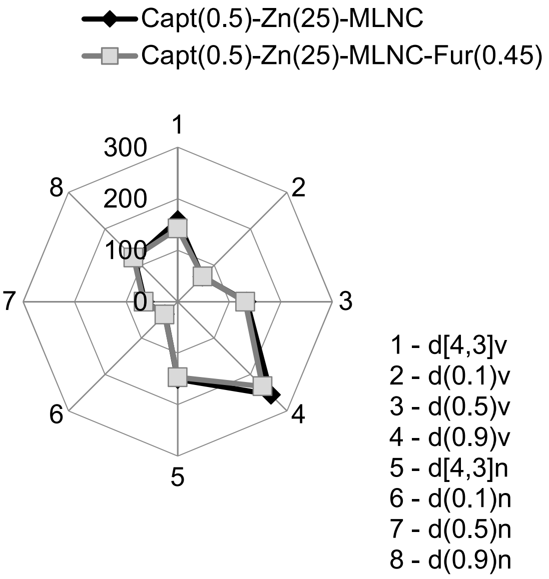

Figure S1. Radar chart of laser diffraction data obtained by volume and by number of particles for Capt(0.5)-Zn(25)-MLNC and Capt(0.5)-Zn(25)-MLNC-Fur(0.45).

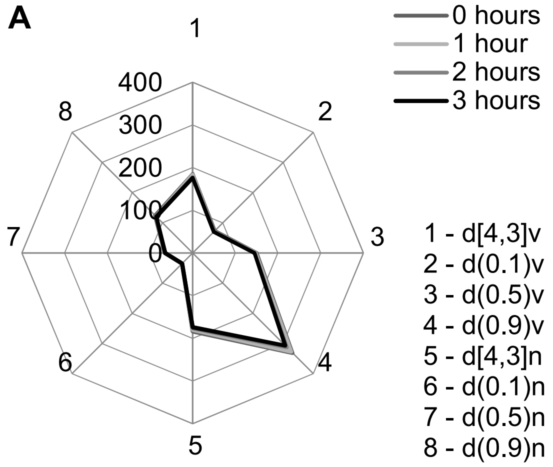

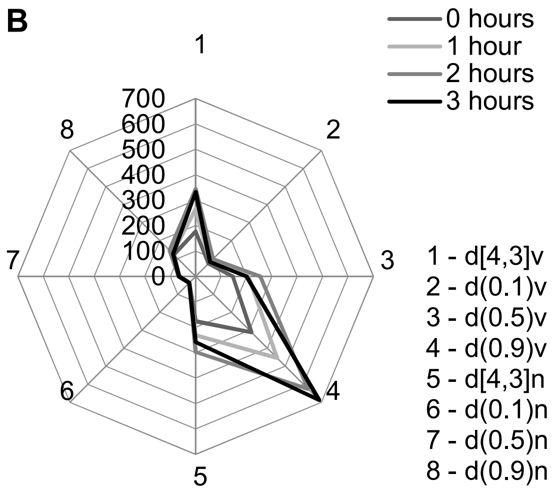

Figure S2. Radar plots of laser diffraction data: **A**) Evolution of particle size distributions as a function of time (0, 1, 2 and 3 hours) after adding Capt(0.5)-Zn(25)-MLNC-Fur(0.45) in simulated gastric fluid (SGF); **B**) Evolution of particle size distributions as a function of time (0, 1, 2 and 3 hours) after adding Capt(0.5)-Zn(25)-MLNC-Fur(0.45) in simulated intestinal fluid (SIF).

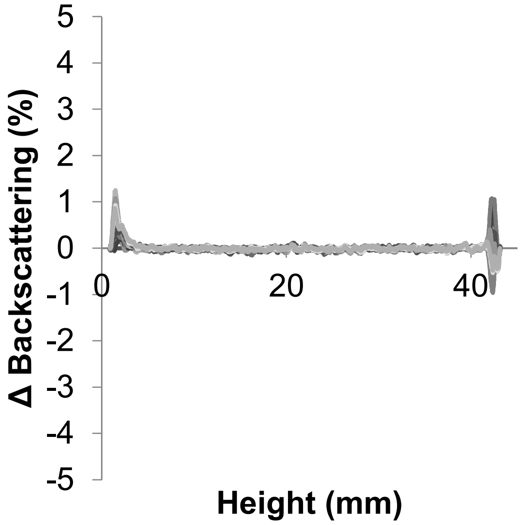

Figure S3. Physical stability evaluation of Capt(0.5)-Zn(25)-MLNC-Fur(0.45) by Multiple Light Scattering.
